# Empirical Evaluation of the Use of Computational HLA Binding as an Early Filter to the Mass Spectrometry-Based Epitope Discovery Workflow

**DOI:** 10.3390/cancers13102307

**Published:** 2021-05-12

**Authors:** Rachid Bouzid, Monique T. A. de Beijer, Robbie J. Luijten, Karel Bezstarosti, Amy L. Kessler, Marco J. Bruno, Maikel P. Peppelenbosch, Jeroen A. A. Demmers, Sonja I. Buschow

**Affiliations:** 1 Erasmus MC, Department of Gastroenterology and Hepatology, University Medical Center Rotterdam, 3015 GD Rotterdam, The Netherlands; r.bouzid@erasmusmc.nl (R.B.); m.debeijer@erasmusmc.nl (M.T.A.d.B.); r.luijten@erasmusmc.nl (R.J.L.); a.kessler@erasmusmc.nl (A.L.K.); m.bruno@erasmusmc.nl (M.J.B.); m.peppelenbosch@erasmusmc.nl (M.P.P.); 2 Proteomics Center, Erasmus MC, Department of Biochemistry, University Medical Center Rotterdam, 3015 GD Rotterdam, The Netherlands; k.bezstarosti@erasmusmc.nl (K.B.); j.demmers@erasmusmc.nl (J.A.A.D.)

**Keywords:** cancer, immunopeptidomics, antigen presentation, HLA-peptide

## Abstract

**Simple Summary:**

Many different human leukocyte antigen (HLA)-types exist across the population that each binds a specific motif of amino acids. HLA-peptide complexes are the driving force behind recognition of cancers and infected cells by cytotoxic T cells. HLA-immunopeptidomics aims to identify peptides derived from (cancer) antigens in the HLA-binding cleft with mass spectrometry (MS). Peptides eluted from HLA are analyzed by MS and translated to a protein derived amino acid sequence by specialized software. These software packages use statistical thresholds to limit false discoveries and return only the most confidently identified peptides. However, we believe, as do others, that many useful peptides can still be found in the excluded pool of peptides. This idea drove the development of specialized algorithms that utilize HLA specific motifs to retrieve additional relevant peptides. It is unknown, however, how many peptides could potentially be found in this pool. By adjusting the statistical threshold, we empirically demonstrate the vastness of valuable data beyond the traditional thresholds that await to be discovered.

**Abstract:**

Immunopeptidomics is used to identify novel epitopes for (therapeutic) vaccination strategies in cancer and infectious disease. Various false discovery rates (FDRs) are applied in the field when converting liquid chromatography-tandem mass spectrometry (LC-MS/MS) spectra to peptides. Subsequently, large efforts have recently been made to rescue peptides of lower confidence. However, it remains unclear what the overall relation is between the FDR threshold and the percentage of obtained HLA-binders. We here directly evaluated the effect of varying FDR thresholds on the resulting immunopeptidomes of HLA-eluates from human cancer cell lines and primary hepatocyte isolates using HLA-binding algorithms. Additional peptides obtained using less stringent FDR-thresholds, although generally derived from poorer spectra, still contained a high amount of HLA-binders and confirmed recently developed tools that tap into this pool of otherwise ignored peptides. Most of these peptides were identified with improved confidence when cell input was increased, supporting the validity and potential of these identifications. Altogether, our data suggest that increasing the FDR threshold for peptide identification in conjunction with data filtering by HLA-binding prediction, is a valid and highly potent method to more efficient exhaustion of immunopeptidome datasets for epitope discovery and reveals the extent of peptides to be rescued by recently developed algorithms.

## 1. Introduction

The action specificity of the adaptive immune system critically depends on the repertoire of peptides presented on human leukocyte antigen (HLA) molecules to T cells [1,2]. As a consequence, rational development of therapy to exploit the adaptive immune system to combat cancer, infection and autoimmune disease, requires insight into which epitopes of which disease-related antigens are presented on HLA. With this purpose, the recent decade has seen an advent of so-called immunopeptidomics, a novel discipline that aims to comprehensively characterize the full complement of peptides presented by HLA complexes to T cells in specific clinical or experimental settings. In immunopeptidomics, cell lines or patient material of interest is typically detergent-lysed and subjected to HLA immunoprecipitation (IP) [3,4]. Peptides are then eluted from HLA at a low pH and analyzed by liquid chromatography-tandem mass spectrometry (LC-MS/MS). This generates MS/MS spectra, which allows the identification of peptides (peptide spectrum matches; PSM) presented by HLA in the original sample. The correct identification of HLA-binding peptides from the obtained MS/MS spectra is currently considered one of the most challenging steps in immunopeptidomics.

Identification of potential HLA-binding peptides from MS/MS spectra can be done in two ways: (1) by matching MS/MS spectra to an experimental and/or in silico generated spectral database (e.g., Mascot [5], Maxquant [6] or PEAKS [7] database (DB) searching-algorithms) or (2) by performing “de novo” sequencing, i.e., reconstructing the amino acid sequence independent of any database, based on the peptide fragmentation pattern (e.g., PEAKS [7], pNovo [8] or Novor [9] de novo algorithms). Identified peptides can subsequently be cross-referenced to existing biomedical literature or subjected to specialized search algorithms that allow the determination of the protein origin of these fragments, even though these peptide sequences may not occur in the reference proteomes [10,11,12]. 

In the first peptide identification approach where raw MS/MS spectra are matched to in silico generated fragmentation spectra, the false discovery rate (FDR) is used as a filter to control the expected proportion of discoveries that are false. The FDR reflects the rate of type I errors expected when testing the null hypothesis in a large dataset. In a typical bottom-up LC-MS/MS-based proteomics workflow, peptides are generated by digestion of proteins with trypsin or another protease. By convention, an FDR of 1% is set by comparing the PSM scores obtained from a database alignment of the experimentally obtained MS/MS spectra to the PSM scores obtained by alignment to a decoy database [13]. However, application of this “standard” FDR threshold may not necessarily be the most efficient for immunopeptidomics for several reasons. First, the databases and PSM score used to derive the FDR threshold were optimized for, and may favor, tryptic peptide identification. While trypsinization of proteins leads to either an arginine or lysine at the peptide C-terminus, HLA-peptides are rather generated by endogenous proteolytic cell processing mechanisms, yielding a wide variety of amino acids at the peptide C-terminus [14,15]. Additionally, since HLA-peptides binding to different HLA-types also differ in their binding properties at the so-called anchoring regions, each immunopeptidome may have its own specific bias towards a certain amino acid composition [16]. Lastly and importantly, in the immunopeptidomics discovery pipeline, LC-MS/MS analysis is followed-up by the selection and further validation of only those peptides that derive from a specific tumor- or pathogen-associated antigen or mutated protein sequence. This selection already greatly reduces the number of hits to investigate and allows for a somewhat less stringent screening approach in the initial stages of the pipeline. In fact, especially for tumor (neo) antigens, immunogenic peptides are rare and validating a few more may sometimes be favored over missing out on potentially curative epitopes. Currently, a range of FDR thresholds have been reported in different immunopeptidomics studies, mostly varying from 1–5% [17,18,19,20,21,22,23,24]. Efforts have been made to develop algorithms that utilize, for example, the binding motifs of HLA-peptides to rescue relevant peptides in the discarded dataset [25,26,27]. These algorithms demonstrated that there are valuable peptides beyond the used statistical thresholds. However, it remains unknown to what extent in general potentially interesting peptides remain below the conventionally used thresholds or how the application of a less stringent FDR affects the resulting peptide set. 

A useful feature of HLA-peptides is that the ligandome of each different HLA-type has preferred (and non-preferred) amino acids at the anchor residues that enable the peptide to bind to that particular HLA-type. This feature lies at the basis for in silico HLA-binding prediction algorithms (e.g., NetMHCcons [28], MHCFlurry [29] or Pickpocket [30]). An LC-MS/MS-derived immunopeptidome would therefore be expected to display a good match between the HLA-type expressed on the cell of origin and the sequence motifs present in the identified peptides [11]. This same principle also underlies the rescue algorithms that utilize HLA-peptide sequence motifs to retrieve motif-containing peptides from discarded datasets [25,27].

Here, using a multitude of HLA-eluates of various origins, we systematically evaluated the influence of varying the FDR threshold during peptide identification on the size of the resulting immunopeptidome and on its content of predicted HLA-binders for the HLA-types expressed on the cells of origin. Our results underscore the common stringent FDR thresholds, although surely yielding the most confident peptide identification, may leave a significant number of potential HLA-peptides undiscovered. In general, our data show that filtering on specific HLA sequence motifs justifies looking for valuable peptides in datasets beyond statistical confidence, which could yield additional epitopes of therapeutic value.

## 2. Materials and Methods

### 2.1. Cell Culture

All cell lines were cultured in RPMI1640, supplemented with glutamine, penicillin/streptomycin and 10% fetal calf serum (FCS; Sigma–Aldrich, St. Louis, MO, USA). Cell lines JY, HepG2, PanC1, MiaPaCa2 and BxPC3 were cultured in T75 or T175 flasks up to ~80% confluency for adherent cells or up to 1–2 × 10^6^ cells/mL as counted by trypan blue exclusion for suspension cultures. Adherent cells were detached with trypsin-EDTA. After harvest, all cells were washed 2–3 times by centrifugation (5 min 450× *g*) with PBS. Primary hepatocytes were isolated from non-tumor tissue obtained from a liver resection. Briefly, the liver tissue was cut into small pieces, treated with collagenase and DNase and subjected to Ficoll density centrifugation to collect a hepatocyte faction that was then washed with PBS, counted and stored at −80 °C in a dry pellet. Usage of this patient material for research purposes was approved by the local ethics committee (MEC2014-060) and the patient provided informed consent.

### 2.2. Sample Preparation, Immunoprecipitations and HLA-Typing

**Figure 1:**
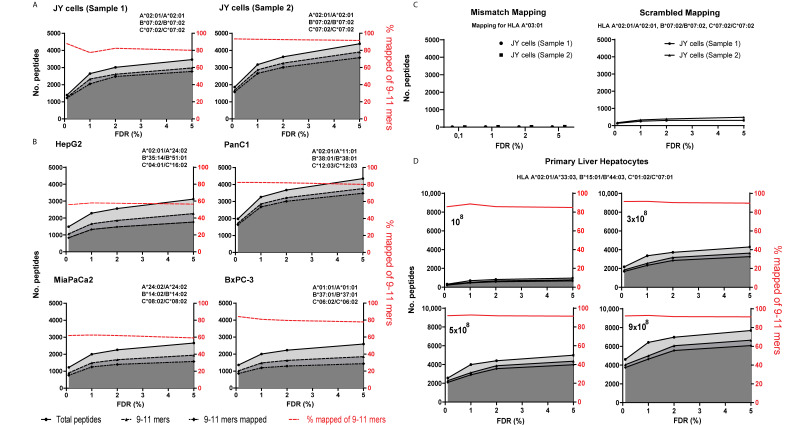
FDR score analysis for various cell lines and primary samples. (**A**,**B**) Obtained immunopeptidomes with the database search of (**A**) JY cells in duplicate and (**B**) various pancreatic and hepatic cancer cell lines. (**A**,**B**,**D**) Shades of grey (top-down) represent the total number of identified peptides, total number of 9–11 mers identified and the total number of 9–11 mers predicted to bind cell- expressed HLA at the indicated FDR (all left *y*-axis). The percentage of predicted HLA-binders of identified 9–11 mers peptides is indicated in red (% mapped on right *y*-axis). (**C**) The left graph shows predicted binding of HLA-derived 9–11 mers to the indicated irrelevant HLA types (mismatch binders) of two independent JY datasets. The right graph depicts the predicted binding of a scrambled peptide dataset containing peptides that are matched in number, length and amino acid composition to peptides derived from two independent JY HLA datasets across indicated FDR thresholds. (**D**) Immunopeptidome of various cell numbers of isolated primary hepatocytes ranked on cellular input from low to high (input number indicated in graph) from left to right and top to bottom. (**A**–**D**) The HLA types used for in silico prediction of HLA-binding are indicated above each graph.

Frozen dry pellets were re-suspended with cold (4 °C) cell suspension buffer (CSB; 50 mM Tris-Cl pH 8 + 150 mM NaCl + 5 mM EDTA) in the presence of one protease inhibitor tablet per 50 mL (complete tablets mini easypack, Roche) to 2 × 10^8^ cells/mL and diluted 1:1 with CSB + 1% Zwittergent 3–12 detergent (*N*-Dodecyl-*N*, *N*-dimethyl-3-ammonio-1-propanesulfonate; Sigma-Aldrich (St. Louis, MO, USA). For cell lysis the suspension was incubated for 1 h on ice and vortexed every 15 min. Subsequently, cell nuclei and large membrane fragments were removed by centrifugation at 17.000× *g* for 10 min at 4 °C to obtain a post-nuclear supernatant (PNS). For IP 100 µL nProtein A fast flow sepharose beads (GE Healthcare) were used either empty (as a pre-clear) or coated with anti-HLA-I monoclonal antibody (mAb); clone W6/32; 3,2 mg antibody/mL packed beads were used to IP HLA class I from PNS. W6/32 mAb was produced in-house from a hybridoma cell line (ATCC). Hybridoma culture medium (without FCS) containing the secreted antibody was pumped over a column of the nProtein A beads. W6/32 was subsequently covalently conjugated to the beads using 20 mM DMP (dimethyl pimelimidate; Sigma-Aldrich, St. Louis, MO, USA) in 0.2 M sodium borate buffer pH 9.0. For all cell lines 100 µL W6/32 coated beads were used for PNS from 10^8^ cells. An exception was made for primary hepatocytes where the number of cells varied as indicated in Figure 1 and only 25 µL beads were used per 10^8^ cells. Both during the pre-clear and subsequent IP, PNS to which beads had been added, was incubated on a roller bench for 1 h at 4 °C. After IP the beads were washed (2 mL per 100 µL packed beads) several times with Tris-NaCl and in the following order with: 20 mM Tris-Cl pH 8.0 + 120 mM NaCl (2×), 20 mM Tris-Cl pH 8.0 + 1 M NaCl (1×), 20 mM Tris-Cl pH 8.0 + 120 mM NaCl (2×), PBS + 20 mM Tris-Cl pH 8.0 (1×) and PBS (1×) prior to peptide elution (described below). For HLA typing purposes, DNA was isolated with a DNA isolation kit (the QIAamp DNA Mini kit; Qiagen 51304) and sent to the Institute for Immunology and Infectious Diseases (Murdoch, Australia) making use of their sequencing based HLA-typing service (NGS illumina-based). 

### 2.3. LC-MS/MS Data Acquisition 

HLA-I peptides were eluted from the beads with 500 µL 0.15% trifluoroacetic acid (TFA) at room temperature (RT). This elution was repeated three times and eluates per sample were combined. The eluted HLA-peptides were lyophilized and stored at −20 °C until mass spectrometry analysis. In order to separate HLA-peptides from contaminating proteins, lyophilized peptides were first dissolved in 400 µL 0.1% TFA and then filtered using a 10 kD MWCO spin column (Amicon 42407). The filtered peptide fraction was desalted using a 1 mL Sep-Pak column containing 10 mg C18 and 10 mg HLB resin that was prepared in-house. Peptides were eluted with 28% acetonitrile containing 0.1% TFA and the solvent was removed by vacuum centrifugation. 

Nanoflow liquid chromatography tandem mass spectrometry (nLC-MS/MS) was performed on an EASY-nLC 1200 coupled to an Orbitrap Lumos Tribrid mass spectrometer (ThermoFisher Scientific) operating in positive mode. Peptide mixtures were trapped on a 2 cm × 100 μm Pepmap C18 column (ThermoFisher Scientific 164564, Waltham, MA, USA) nand then separated on an in-house packed 50 cm × 75 μm capillary column with 1.9 μm Reprosil-Pur C18 beads (Dr. Maisch) at a flow rate of 250 nL/min, using a linear gradient of 0–32% acetonitrile (in 0.1% formic acid) over 2 h. Mass spectra were acquired from 375 to 1200 m/z in the Orbitrap at 120,000 resolution. Upon selection, peptides were fragmented by higher-energy collisional dissociation (HCD) with a collision energy of 30% and MS/MS spectra were recorded in the Orbitrap at 30,000 resolution. 

### 2.4. Bioinformatics Analysis 

Mass spectrometry data were analyzed with PEAKS Studio v 10.5 (Bioinformatics Solutions Inc. Waterloo, ON, Canada). MS/MS spectra were searched against a database containing sequences downloaded from UniProt for H. sapiens (version August 2019). The digest mode was set to “unspecific” (no enzyme), error tolerances for parent mass and fragment masses were 10.0 ppm and 0.02 Da, respectively. The peptide FDR varied from 0.1 to 5%. NetMHCcons v1.1 (DTU Bioinformatics [28]) was used to predict HLA-binding properties of peptides to HLA-types of interest. A peptide was called an HLA-binder at a predicted IC50 ≤ 500 nM or rank score ≤ 2%. HepG2 proteome data were downloaded from a mass-spec characterization study [31]. Extracted proteins were ranked based on quantification from high to low expression based on the average of their three measurements for HepG2. A complimentary list was generated by extracting all source proteins (obtained via PEAKS, by accession numbers) from our immunopeptidome data. The protein list from the Geiger et al. study [31] was taken as the leading list and every time a protein was present in our immunopeptidome list, it received a score of 1. GraphPad Prism was used to generate plots and barcode figures. 

## 3. Results

### 3.1. Experimental Data Set

To test the effect of various FDR values on the size of the immunopeptidome and number of bona fide HLA-binders, we performed an extensive immunopeptidome analysis on HLA-eluates of various cell lines and primary cell samples (Figure 1). We included five different cell line models in this study: in casu JY cells, a professional antigen presenting leukemic B cell line often used for immunopeptidomics studies; HepG2 cells, a model liver hepatoma cell line that represented liver cancer which is often considered an attractive target for therapeutic vaccination; and three different pancreatic cancer cell lines (PanC1, MiaPaCa2, and BxPC-3), representing an oncological disease that is usually considered to be very challenging with regard to immunotherapy. All five experimental models were expanded to a final experimental size of 10^8^ cells, after which cells were lysed and HLA was immunoprecipitated (see methodology). Typically, 50–70% of all HLA complexes were retrieved in this procedure (data not shown; determined by Western blot analysis as the relative HLA signal retrieved by IP compared to the input material). 

### 3.2. More Permissive FDR Settings Improve Coverage of the Immunopeptidome

We next assessed the effect of different FDR thresholds in a mass spectrometry proteomics based database search using the PEAKS database (DB) search algorithm. Figure 1 shows the resulting identified peptide sets for different cell lines and different FDR value thresholds. As HLA-I-bound peptides typically contain nine to eleven amino acids (9–11 mers), we subsequently selected only 9–11 mers that were identified from fragmentation spectra (the m/z detection window of the mass spectrometer was also limited to this range, see methodology). To gain further insight into the probability that an identified peptide would have been retained in the peptide-binding groove of the HLA molecules expressed on the cell of origin, we predicted HLA-binding strengths for each peptide sequence. For this, we used the MHCcons 1.1 software tool [28] utilizing the most commonly applied binding criteria (i.e., IC50 ≤ 500 nM or rank score ≤ 2%; see methods). For all five cell lines that expressed widely divergent HLA-types (indicated in Figure 1), we observed that increasing the FDR threshold increased putative peptide identifications and that the majority of these additional identified HLA-peptides were invariably also predicted to bind the HLA-types expressed on the cell of origin (stable red lines in Figure 1). Thus, the application of a less stringent FDR threshold results in an overall increased yield of potential HLA-peptides. This effect was tested and observed for FDR values of up to 5% (Figure 1A,B). 

The identification of the immunopeptidome from JY cells was performed in duplicate. Duplicates yielded very similar results underscoring the reproducibility of our analysis (Figure 1A). To further test the specificity of the in silico HLA-binding prediction tool, we also predicted binding to an irrelevant HLA-type for all cell lines (Figure 1C for JY and Supplementary Figure S1 for the other cell lines). For the JY sample, for example, identified peptides were mapped to HLA A*03:01, which is a mismatch for A*02:01 (full HLA-type of JY cells depicted in Figure 1A). The prediction to irrelevant HLA-types yielded only a low number of predicted binders (<3% of 9–11 mers), even at higher FDR thresholds, indicating that the identified peptides were indeed specific binders exclusively for HLA-types expressed on the source material. Then, to also rule out any specific prediction results based on the amino acid content of our dataset, we randomly generated a database of 9–11 mers peptide sequences with an identical total number of peptides and identical length and amino acid distribution to each of the datasets obtained with the different FDR thresholds (i.e., scrambled). The HLA-binding prediction for these scrambled sequences using the netMHCcons 1.1 tool resulted in only very few predicted HLA-binding sequences (Figure 1C).

To subsequently assess the sensitivity of the HLA-binding prediction to peptide misidentifications we evaluated how the in silico prediction of HLA-binders would perform on a peptidome generated by de novo sequencing. In PEAKS, the probability that a peptide is correctly identified using the de novo sequencing algorithm is indicated by the average local confidence (ALC) score. Peptides identified with higher ALC scores are more likely to be identified correctly and decreasing the permitted ALC score is expected to result in more falsely identified peptides, which in turn can be expected to affect predicted HLA-binding. Indeed, we found a direct inverse relationship between the ALC score and the number of correctly predicted HLA-binders (Supplementary Figure S2). This pattern contrasted with the stable high percentages of correctly predicted HLA-binders that were observed at less stringent FDR values, suggesting that the latter represent bona fide HLA-binders (based on the prediction algorithm). Together, these additional controls support the idea that releasing the FDR threshold for HLA-peptide discovery combined with HLA-binding prediction is a valid approach. 

Identification of more targets from existing immunopeptidome datasets could benefit target discovery and subsequent vaccine design, which are of large interest in the field of oncology. Our results imply that there may be false negative identifications of peptides when stringent FDR values are used in peptide database searching algorithms. To illustrate this we therefore searched for peptides from tumor associated cancer/testis antigens (CTA) in the cell line-derived immunopeptidomes obtained using variable FDR cutoffs. A higher number of CTAs were identified using increasing FDR cutoffs (Supplementary Table S1), although the highest gain was observed when increasing the FDR threshold from 0.1% to 1%, yielding 6 and 17 CTA-derived HLA-peptides, respectively. One additional CTA-derived peptide was added when further releasing the FDR threshold to 5%. 

### 3.3. Immunopeptidomic Analysis of Variable Amounts of Primary Cells Yielded Similar Results

Our results so far were obtained in transformed cell line models displaying uncontrolled growth. It is widely recognized that antigen presentation on HLA molecules may be markedly different in such model systems as compared to untransformed primary cell types. Hence, it is of interest to validate our findings also on primary cells. Thus, we extended our analysis to primary hepatocytes and also included a titration of cell input to explore the dynamics across peptide abundancy levels. In line with our expectation, it was observed that the amount of cells highly affected the overall number of uniquely identified peptides. Importantly, for all samples irrespective of cellular amounts, increases in peptide yield were observed as a consequence of releasing the FDR threshold and again the relative number of predicted HLA-binders remained stable (red line Figure 1D). Our results thus suggest that the potential to discover additional HLA-peptides at higher FDR thresholds is a general property of antigen presenting systems. 

Next, we reasoned that less abundant peptides can be expected to have a lower quality spectrum and therefore may be less likely to be identified when applying relatively low FDR thresholds. To test this, we investigated the effect of increasing cellular input on the identification of low quality peptides. We first isolated the predicted HLA-binders from the 10^8^ cell sample that were identified in the FDR range of 1–5% (174 peptides). Subsequently, we looked for these specific peptides in the sample with a higher input of 9 × 10^8^ cells. Strikingly, 150 of the 174 HLA-binders (86.2%) were identified in this high input sample when applying a more stringent FDR value of 1%. Moreover, when we extended our search to an FDR of 1–5% we found an additional 13 peptides back. Altogether, the majority of the predicted HLA-binders with poorer spectra in the low input sample could be found back at a stricter FDR in the high input sample, likely due to more robust peptide spectra as a result of higher peptide abundance.

### 3.4. Comparing Immunopeptidomic Results to Full Cellular Proteomes

**Figure 2:**
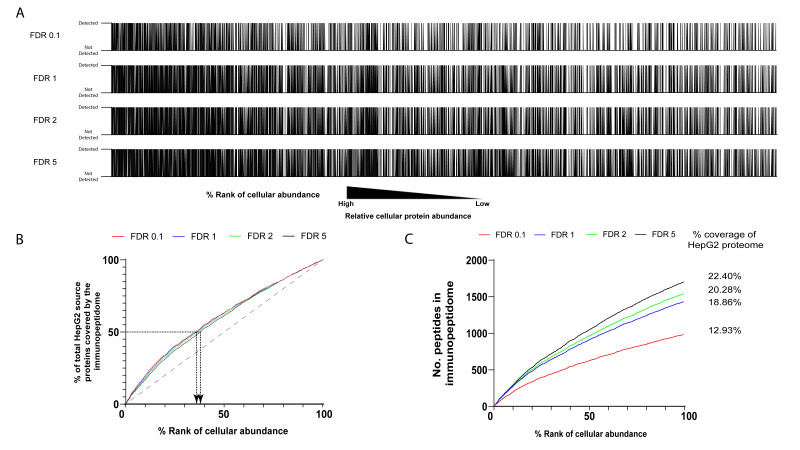
Comparison of our HepG2 immunopeptidome to the quantitative HepG2 cellular proteome: (**A**) proteins from the HepG2 proteome were sorted by cellular expression from high (left) to low (right). Then source proteins in this list for which one or more 9–11 mer peptides were identified in the HepG2 immunopeptidome, using indicated FDR cutoffs, were marked by a vertical line to yield barcodes. (**B**) While “walking” from left to right over these barcodes a cumulative score was calculated by adding a 1 for each protein hit in the immunopeptidome. At each position in the abundance ranked protein list (*x*-axis) this cumulative score was then plotted as a percentage of the total of proteins covered by the immunopeptidome and also by the full HepG2 proteome (*y*-axis). Arrows indicate the % of top ranking source proteins that produced 50% of HLA-peptides. (**C**) As in B but representing the cumulative absolute number of proteins covered by the immunopeptidome at each cellular abundance rank. Indicated on the right is the percentage of proteins in the HepG2 proteome from which peptides were identified in the immunopeptidome.

Previously, others have demonstrated that peptides derived from more abundant proteins are also more frequently identified in immunopeptidomes [32]. If more abundant proteins are indeed more frequently presented on HLA, these may yield better PSM scores upon MS/MS analysis of HLA-eluates favoring their identification at a more restrictive FDR as exemplified by our primary hepatocyte titration result. However, peptide loading on HLA is a complex process, which also involves competition between peptides depending on their binding affinity and half-life, as well as other factors including peptide generation and degradation kinetics. This means that theoretically the HLA molecule may not necessarily favor only the peptides from the highest expressed proteins. To test the relation between cellular protein abundance and the number of HLA-peptides identified from a protein, we mapped our immunopeptidome of HepG2 cells to a publicly available quantitative proteome dataset from this same cell line [31]. We then ranked the relative cellular abundances of HepG2 proteins from high to low and marked those proteins for which one or more peptides were identified in our HLA peptidome (*x*-axis in all panels in Figure 2). This yielded a binary barcode graph visualizing the relation between the presentation of a protein in HLA and its reported cellular abundance (Figure 2A). Indeed, most lines representing peptide identifications in our immunopeptidome clustered on the left side of the bar code, indicating that they originated from highly abundant cellular proteins. We generated similar plots across FDR thresholds to visualize the effect of applying different FDR values on the abundance distribution of HLA-peptide source proteins, finding additional hits in the lower abundant proteins (on the right side) in case of more lenient FDR values (Figure 2A). The effect of varying the FDR thresholds, however, was hard to discern visually. To obtain a more quantitative assessment of enriched peptides from highly abundant source proteins in our HLA peptidome, a cumulative score was calculated by walking from highest abundant protein to lowest abundant protein and adding a score of 1 every time a HepG2 protein was encountered in our converted (from peptide to protein) immunopeptidome dataset. This cumulative score was then plotted at each position of the abundance ranked protein list as a proportion of the HepG2 cell proteome covered in our immunopeptidome (Figure 2B). If HLA-peptides would derive equally frequently from all proteins along the abundance spectrum, an exact diagonal line would be expected (Figure 2B; broken line). Preference for peptides to derive from more abundant proteins would deviate the graph upwards. We observed that the immunopeptidomics dataset for all FDR values favored higher abundant proteins (Figure 2B). Only small differences were observed between the application of an FDR of 1% or 5%. At an FDR of 1% half of the presumed HLA-peptides in the dataset derived from the top 35.94% of the most abundant proteins (Figure 2B; left arrow). Using an FDR value of 5%, however, half of the detected peptidome derived from the top 37.93% of the most abundant proteins (Figure 2B; right arrow). At an FDR threshold of 5% hundreds of additional source proteins were detected in the immunopeptidome including some more moderately expressed in the cell. The total coverage of the HepG2 proteome in the immunopeptidome dataset was 18.86% (of reported HepG2 proteins) at an FDR of 1% vs. 22.40% at an FDR of 5% (Figure 2C). Vice versa at the FDRs of 1% and 5%, respectively, 70.79% and 69.66% of the here identified HLA-peptides mapped to reported HepG2 proteins. Together with the HLA-binding results, this stable amount of HLA-peptides that derive from proteins present in the HepG2 proteome, provides further indication that the additional peptides identified at high FDR thresholds are likely true positives. Taken together, our results confirm previous findings that most detected HLA-peptides are derived from more abundantly expressed cellular proteins irrespective of the FDR threshold used but indicate a slight deviation towards less abundant proteins at more permissive FDR thresholds. 

## 4. Discussion

**Figure 3:**
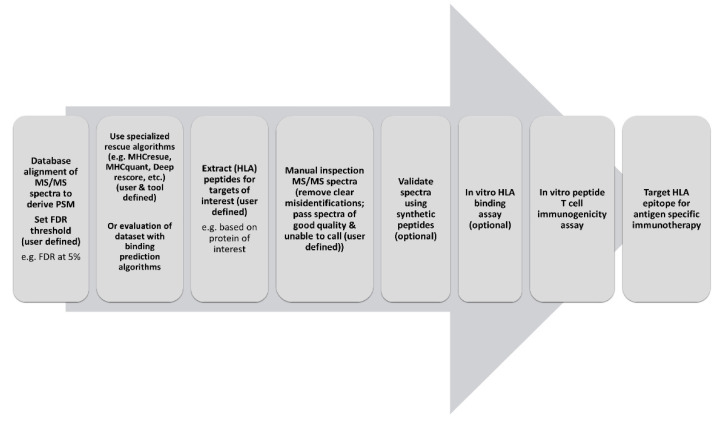
Proposed workflow regarding the use and handling of mass spectrometry data in the application and discovery of HLA-peptides to be used for antigen-specific immunotherapy.

Understanding the nature of the antigen repertoire presented to the adaptive immune system is essential for better treatment of cancer and autoimmune disease, but is technically challenging. Although important progress in the field of HLA-immunopeptidomics has been made [4], many questions remain. An important realization is that the standardized approaches to analyze the cellular proteomes, i.e., bottom-up tryptic proteomics, can only be partially transposed to the analysis of the immunopeptidome. The present study adds to this notion by evaluating whether commonly used FDR thresholds in bottom-up proteomics are optimal for immunopeptidome analysis using the theoretical property of predicted HLA-binding for quality assessment. For bottom-up tryptic proteomics an FDR of 1% is the widely accepted standard [13]. However, we show that a less stringent FDR threshold yields a larger collection of PEAKS peptide identifications. This finding is in line with results of reported rescue algorithms exploiting the concept that MS identified peptides should contain a binding motif for one of the specific HLA-types expressed in the cells of origin [25]. Our data thus provide further rationale for such strategies to uncover additional peptides of potential interest for epitope discovery. However, overall quality of spectra of peptide identifications in the FDR range of 1–5% was found reduced (based on expert opinion), despite their predicted HLA-binding, but in agreement with their inherent lower PSM scores. This leaves us with the challenge of how to deal with putative peptide identifications that contain a binding motif of the corresponding HLA-type, but harbor too poor spectra for manual validation. Although expert opinion is not to be neglected, HLA-binding prediction may still render peptides with poorer unevaluable spectra of interest for epitope discovery. This is especially valuable when such a peptide is derived from a specific protein of interest, for example in a study trying to acquire potential epitopes for a vaccine against a certain tumor associated- or pathogen-derived protein. Although beyond the scope of the current study, additional evidence for correct peptide identifications can be obtained by using synthetic forms of discovered PSMs to validate their identification by MS identification, in vitro HLA-binding confirmation and immunogenicity assays (Figure 3). Such a workflow may grant an efficient trade-off between the ends of the sensitivity and specificity spectrum. At the end of maximal specificity, the application of an FDR of 1% without additional HLA-binding algorithms, acquiring limited data filtered only for the peptides with the highest technical quality, but possibly missing valuable data in a discovery setting. On the other end of the spectrum, optimal sensitivity can be reached by not applying any statistical thresholds to control the size of the dataset, capturing all the potentially valuable data, but likely also many false hits. Our data support a workflow that combines the best of both worlds by releasing the first FDR filter but adding a second filter specific to this field of research extracting only HLA-binding peptides to keep the amount of data manageable and reliable (Figure 3). This could be done manually by utilizing HLA-binding algorithms and setting binding parameters for known HLA-types expressed in the source material. However, great efforts have been made to develop algorithms that directly implement the binding motifs of HLA-peptides identified at high confidence to distill peptides harboring this same motif from beyond the set FDR threshold [25]. Peptides derived from specific proteins of interest or from mutated protein sequences can be subsequently extracted from the dataset for further validation. In this scheme, we propose to restrict manual inspection of spectra to peptides of specific interest and possibly only to call certain misidentifications. Unevaluable spectra of peptides that are predicted to bind donor HLA, however, may still be considered to be followed-up. 

In the present study, an FDR threshold of 5% was the most permissive FDR analyzed and this threshold still delivered peptides equally well predicted to bind to HLA as those obtained using lower FDR threshold cutoffs. Here, it should be noted that HLA-binding was a theoretical assessment that for translation to immunological relevance remains to be validated in vitro. It is unclear whether even less strict FDR thresholds would still provide more opportunity. The PEAKS software allows for easy pre-set data filtering up to a threshold of FDR 5%. Although it may be possible to set custom thresholds beyond this level, we have chosen not to pursue this further because in that range the data will be increasingly contaminated with false hits and the size of the data would rapidly take on less manageable proportions. Thus we limited our study to an upper FDR threshold of 5% but acknowledge there may be remaining potential beyond this limit that can be explored.

From primary hepatocyte HLA, peptides could be found back with a higher amount of cells at a more restrictive FDR, suggesting that more cells will support the discovery of additional peptides. On the other hand, often a limited number of cells are available. As we have shown here that the majority of identified peptides at more lenient FDR settings can still be found back with a stricter FDR at a higher input, the release of FDR in these situations certainly deserves consideration. Our findings demonstrate the ability and power of the peptide spectrum match algorithm to identify these peptides even at lower abundances. While rescue algorithms [25,26,27] can capture peptides beyond the set statistical threshold of confidence, experiments with primary hepatocytes argue that using more cells contribute to a better profile and a more complete dataset. The maximum amount of cells/HLA-input for complete data capture remains to be determined but is consequently more likely reached when lower confidence peptides can also be added to the equation.

Others have previously found that HLA-peptides preferably derive from the most abundant cellular proteins and those with the highest turnover [32]. Interestingly, usage of a more restrictive FDR threshold seems to favor detection of HLA-peptides derived from more abundantly expressed source proteins, possibly also suggesting a higher abundance of these peptides in our peptidome and associated better spectra. One could argue that the increased source protein coverage and the slightly more widespread distribution over the abundance spectrum of source proteins of peptides derived using a more permissive FDR, point to a higher level of false identifications. However, primary hepatocyte data shows that a majority of peptides discovered at permissive FDR settings can also be found with an increased input of cells with a stricter FDR threshold. Furthermore, these additional HLA-peptides identified at more permissive FDR settings equally bind source cell HLA-types. For these peptides to still be false positive hits, they would need to contain the correct amino acid motif to pass the filter of the HLA mapping which we believe unlikely to occur at a high rate due to chance. This is supported by the results of our control experiments predicting binding of peptides to irrelevant HLA-types and using scrambled matched datasets as input for HLA mapping. In addition, considering the sensitivity of HLA-prediction to sequence uncertainty by de novo sequencing, we believe that the amount of false positive peptides after HLA-mapping is likely small. 

Although the application of HLA-binding to curate the immunopeptidome can salvage peptides identified at lower confidence, including HLA-binding will also introduce a bias. Weaker HLA-binders might be missed at the fixed threshold on the IC50 or rank score during in silico prediction, as will bulging or overhanging peptides with a deviating length. In this study, peptide length was limited to 9–11mers as these are the most common HLA-peptide lengths. 

Our observations strengthen our confidence in the validity of applying a workflow of combining a (more permissive) FDR filter with an HLA-binding filter as proposed (Figure 3). The decision to use stricter (e.g., 1%) or more permissive (e.g., 5%) FDR thresholds may need to be tailored to the situation taking into account tissue availability, the scarcity of target epitopes options, the manageable number of peptides to validate with downstream assays and lastly the goal of the study. For example, in a scenario with little tumor material and peptide hits, a more permissive FDR could be favored while with plenty of tissue and target peptide hits available a more restrictive choice can be made. 

Taken together, our study supports that guided by in silico HLA-binding calculations, FDR thresholds used to identify peptides from HLA-eluates can be used in a more permissive manner to yield more potential HLA-binders for usage in antigen specific immunotherapeutic approaches such as vaccines or adoptive T cell transfer.

## 5. Conclusions

The empirical evaluation of computational HLA-binding in this study revealed that beyond the traditionally used statistical threshold, relevant and valuable data could still be distilled by applying an HLA-binding motif based filter. Altogether we conclude that the use of data beyond conventional statistical thresholds retrieved by specialized algorithms or in silico prediction tools are justified to enhance the coverage of the immunopeptidome.

## Data Availability

The data presented in this study are available on request from the corresponding author. The data are not publicly available due to privacy and ethical reasons.

## Supplementary Materials

The following are available online at https://www.mdpi.com/article/10.3390/cancers13102307/s1: Figure S1: mismatch mapping analysis of cell lines used in this study, Figure S2: ALC score analysis of de novo acquisition of cell lines used in this study, Table S1: overview of identified cancer testis antigen peptides from cell lines.
