# Supplementary material for: Empirical Evaluation of the Use of Computational HLA Binding as an Early Filter to the Mass Spectrometry-Based Epitope Discovery Workflow
Source: Cancers (Basel). 2021 May 12;13(10):2307. doi: 10.3390/cancers13102307 (PMC8150281; doi:10.3390/cancers13102307)
Supplement: Supplementary file 1 [file cancers-13-02307-s001.zip › cancers-1173566-supplementary.pdf]

Empirical Evaluation of the Use of Computational HLA Binding as an Early Filter to the Mass Spectrometry-Based Epitope Discovery Workflow

Rachid Bouzid, Monique T. A. de Beijer, Robbie J. Luijten, Karel Bezstarosti, Amy L. Kessler, Marco J. Bruno, Maikel P. Peppelenbosch, Jeroen A. A. Demmers and Sonja I. Buschow

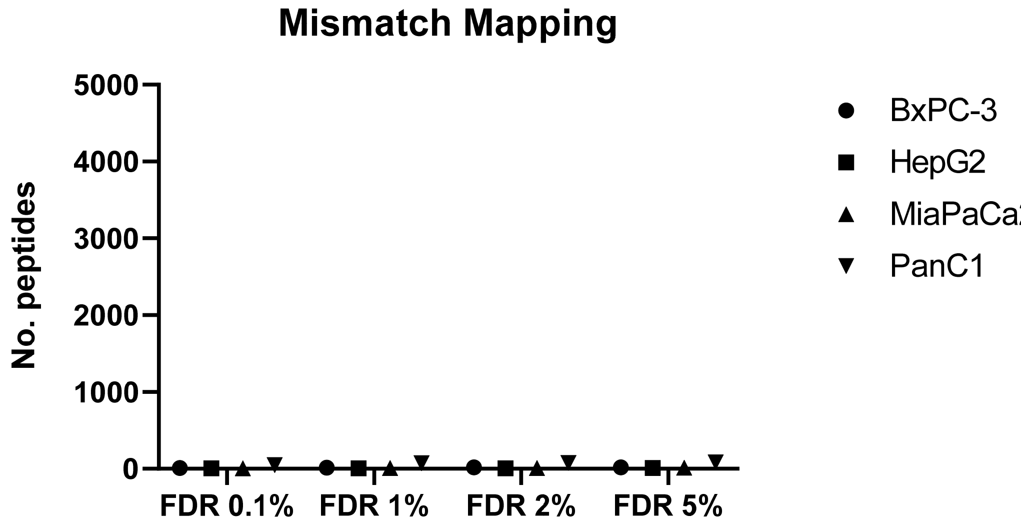

Figure S1. Peptide datasets from various cell lines were tested for containment of random peptides by the mapping of an irrelevant HLA-type; HLA-A*01:01 (for PanC1), HLA-A*02:01 (for BxPC-3) and HLA-A*03:01 (for MiaPaCa2 and HepG2).

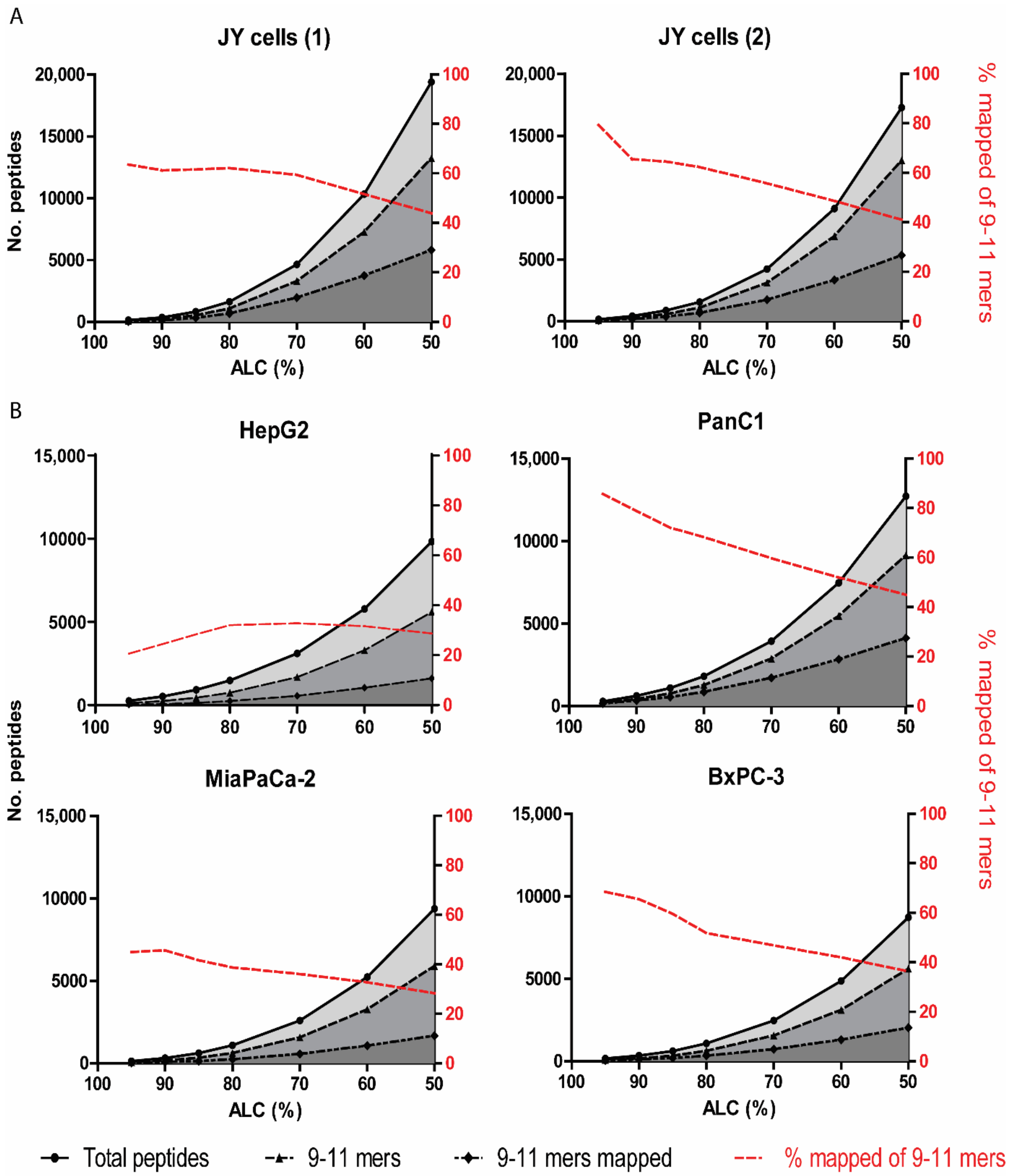

Figure S2. ALC score analysis for various cell lines and samples. (**A**–**B**) Inventory obtained immunopeptidomes of indicated model cell lines (**A**) JY cells in duplicate and (**B**) various pancreatic and hepatic cancer cell lines. (**A**-**B**)In shades of grey (top-down) the total number of identified peptides, total number of 9-11mers identified and the total number of 9-11mers predicted to bind cell expressed HLA at the indicated FDR (all left *y*-axis). In red the percentage of predicted HLA-binders of identified 9-11mer peptides (% mapped on right *y*-axis).

Table S1. Overview of identified cancer testis antigen peptides from cell lines.

Cell line	Peptide	FDR 0.1	FDR 1	FDR 2	FDR 5	CTA
PanC1	QHSDKIIRL	x	x	x	x	TTK
	SVPLSDALLNK		x	x	x	TTK
	SLLQHLIGL		x	x	x	PRAME
	IHSSIVSTL		x	x	x	ATAD2
	SVQKPKFPK		x	x	x	CASC5
	AVIDNFVKK		x	x	x	DDX43
BxPC3	EVDPIGHLY	x	x	x	x	MAGEA3
	DPIGHLY		x	x	x	MAGEA3
MiaPaCa-2	KYLTVKDYL	x	x	x	x	ATAD2
	LYPEVFEKF	x	x	x	x	ATAD2
	AYAIIKEEL	x	x	x	x	ATAD2
	TLDFENEKL	x	x	x	x	CEP55
	YSDDDVPSV		x	x	x	ATAD2
	VYTLDIPVL		x	x	x	ATAD2
	VYVDDIYVI		x	x	x	CASC5
	IYVIPQPHF		x	x	x	CASC5
	NIDINNNEL		x	x	x	CASC5
	AIIKEEL				x	ATAD2

Comparison of detection of CTAs as listed in the CTDatabase of The Ludwig Institute for Cancer Research (http://www.cta.lncc.br/; accessed on the 22nd of July 2020) at various FDRs.
